# Enhanced rehabilitation guidance after arthroscopic capsulolabral repair of the shoulder: a randomized controlled trial

**DOI:** 10.1177/0269215520919472

**Published:** 2020-05-07

**Authors:** Juhani Multanen, Pauli Kiuru, Kirsi Piitulainen, Jari Ylinen, Juha Paloneva, Arja Häkkinen

**Affiliations:** 1 Faculty of Sport and Health Sciences, University of Jyväskylä, Jyväskylä, Finland; 2 Department of Physical Medicine and Rehabilitation, Central Finland Hospital District, Jyväskylä, Finland; 3 Department of Surgery, Central Finland Hospital District, Jyväskylä, Finland; 4 School of Medicine, University of Eastern Finland, Kuopio, Finland

**Keywords:** Shoulder operation, shoulder instability, shoulder exercise, long-term follow-up, American Shoulder and Elbow Surgeons Standardized Shoulder Assessment Form

## Abstract

**Objective:**

To compare the effects of a 12-month home-based exercise program to usual care in patients after arthroscopic capsulolabral repair of the shoulder.

**Design:**

Randomized controlled trial.

**Setting:**

Outpatient physical and rehabilitation medicine clinic.

**Subjects:**

Forty-five patients (mean age: 35 years; standard deviation (SD): 10 years) who underwent arthroscopic capsulolabral repair due to labral lesion were randomized into an exercise group (EG) or a control group (CG).

**Intervention:**

The EG received a 12-month home-based additional exercise program with four physiotherapy follow-up visits, while the CG received standard postoperative exercise instructions.

**Main measures:**

Self-reported shoulder disability was assessed with the American Shoulder and Elbow Surgeons Standardized Shoulder Assessment Form (ASES) and quality of life with the Short-Form (SF)-36 Health Survey. The function of the operated shoulder was evaluated with strength and range of motion measurements.

**Results:**

No between-group differences were observed in any of the outcomes at the follow-up. Mean ASES score improved by 16 (95% confidence interval (CI): 10–23) points from the baseline 78 (SD: 17) in the EG and 13 (95% CI: 7–19) points from the baseline 79 (SD: 17) in the CG. Both groups achieved a significant improvement in the dimensions of Physical Functioning, Role-Physical, and Bodily Pain of the SF-36 and in every aspect of strength and range of motion measures. In EG, exercise adherence was moderate (52%) during the first six months and poor (22%) during the last six months.

**Conclusion:**

Home-based additional exercises with four outpatient follow-up visits did not improve outcome after arthroscopic capsular repair of the shoulder.

## Introduction

Arthroscopic anterior capsulolabral repair is a surgical procedure commonly used in the treatment of anterior shoulder instability associated with a Bankart lesion.^1^ There is evidence to show that a surgery including postoperative rehabilitation may be more effective in reducing shoulder dislocations than non-operative rehabilitation alone.^2^ However, only few high-quality studies have investigated the effect of various rehabilitation protocols on functional outcome after arthroscopic anterior shoulder stabilization.^3–5^ Kim et al.^3^ found no differences between an accelerated and a conventional rehabilitation protocol for self-reported disability or recurrence rate in dislocation in their randomized controlled trial. Patients were followed-up for a mean of 31 months. Ismail and El Shorbagy,^4^ in their randomized controlled trial for self-reported disability, similarly reported finding no differences between a supervised and home-based rehabilitation protocol after 24 weeks. Damkjaer et al.^5^ compared patient cohort receiving rehabilitation according to the guidelines issued by the American Society of Shoulder and Elbow Therapists to those receiving standard care and found no between-group differences in self-reported disability, quality of life, and range of motion.

Because of the paucity of randomized controlled trials finding effective rehabilitation protocol after arthroscopic anterior shoulder stabilization, further research is needed to optimize patient outcome. In addition, patient adherence to exercise protocols has been inconsistently described in the postoperative rehabilitation literature. Good adherence to exercise and physical activity may improve long-term effectiveness. However, despite the known benefits of exercise, training adherence typically diminishes over time.^6^ It has been proposed that the addition of refresher sessions could improve adherence to exercise regimens and hence improve clinical outcomes in different musculoskeletal disorders.^7^

Thus, in the present study, we sought to explore whether a long-term home-based exercise program would improve subjective self-reported physical function, quality of life, and objectively measured shoulder function over a 12-month follow-up after arthroscopic anterior shoulder stabilization. We hypothesized that patients performing additional home-based exercises and randomized to receive physiotherapy control visits would show greater improvement in self-reported physical function, quality of life, and objectively measured shoulder function than those performing home exercises without control visits, that is, receiving usual care.

## Methods

**Figure 1.**
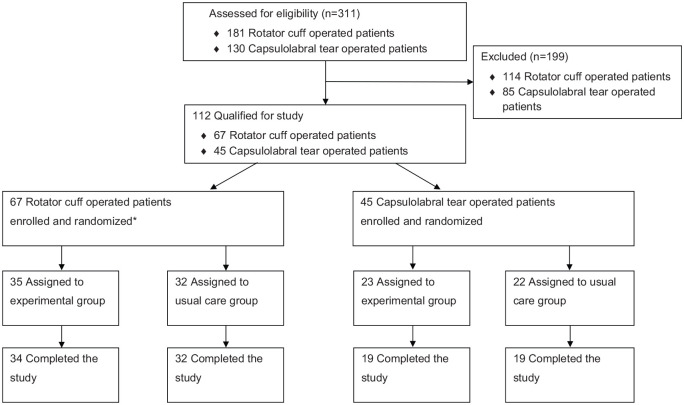
Enrollment, randomization, and retention of the ClinicalTrials.gov Identifier: NCT00624117 “Progressive Exercise After Operation of Rotator Cuff Rupture and Anterior Labrum Rupture.” Source: Published in *Clin Rehabil*. 2015 May; 29(5): 447–456.

The study is registered in the ClinicalTrials.gov database (NCT00624117; Scientific title: “Progressive Exercise After Operation of Rotator Cuff Rupture and Anterior Labrum Rupture”). The trial registration included two trials (Figure 1). In this article, we provide data from the postoperative exercise program after surgical treatment of anterior labrum rupture. We have presented earlier data from the trial examining the shoulder surgery and the subsequent postoperative exercise program of the rotator cuff rupture.^8^

This randomized controlled trial was conducted between May 2006 and December 2009. The study was approved by the Ethics Committee of the Central Finland Health Care District with the diary number Dnro46/2005. The participants provided their written consent according to the Helsinki Declaration. The outpatient clinic of the Department of Physical Medicine and Rehabilitation in Central Finland Hospital was responsible for the integrity and conduct of the study. Originally, the execution of the study was supported by the Medical Research Foundation of the Central Finland Health Care District. The funder had no role in study design, data collection and analysis, decisions to publish, or preparation of the manuscript. We used the CONSORT Statement in designing and reporting this randomized controlled trial.

Participants for this 12-month trial, comparing enhanced postoperative rehabilitation program and usual care, were recruited from patients referred for capsulolabral repair surgery in the Department of Orthopedics and Traumatology in Central Finland Hospital. All consecutive candidates for the procedure were informed about the postoperative rehabilitation program, and their preliminary eligibility was assessed with the following inclusion criteria: anterior capsulolabral Bankart lesion, age between 18 and 55 years, and willingness to undergo arthroscopic shoulder surgery and participate in the subsequent randomized postoperative rehabilitation trial. Exclusion criteria were previous surgery on the affected shoulder, posterior glenohumeral instability, cervical intervertebral disk prolapse, previous operations on the cervical spine, stenosis of the cervical spinal canal, signs of marked osteoarthritis, rheumatoid arthritis, fibromyalgia, pregnancy, a serious mental illness or social problems, a severe disease or neurological disorders, or difficulty in understanding the Finnish language. Patients who met the inclusion criteria and indicated their willingness to participate to the postoperative rehabilitation program by knowing that they had an equal chance of being assigned to either the enhanced rehabilitation group or usual care group underwent an arthroscopic capsulolabral repair between May 2006 and December 2008.

After the operation all patients followed the same rehabilitation protocol for the first two months. The operated arm was maintained beside the body in a suspension sling for three weeks, although patients were also allowed to perform light household activities without the sling during this period.

Patients were instructed by a physiotherapist in the postoperative home exercise protocol, which was to be performed three times per day. Exercises included active flexion and extension of the elbow and fingers, retraction of the scapula and shoulder, pendulum exercises, passive or assisted shoulder flexion, shoulder external rotation to 60°, and functional internal rotation of the shoulder. These exercises were started on the first day after the operation.

All patients saw a physiotherapist two weeks after the operation during a routine visit to the outpatient clinic of physical and rehabilitation medicine. Light isometric contractions of the shoulder muscles (in flexion, extension, internal and external rotation) were added to the patients’ normal postoperative rehabilitation protocol. These isometric contractions were to be done three times per day.

Six weeks after the operation, each patient visited the outpatient clinic again and was advised to start exercise using a light resistance bands (yellow-colored Thera-Band^®^; The Hygenic Corporation, Akron, OH, USA), which they received for free and dynamic range of motion exercises without extra resistance. The resistance exercises were to be performed two to three times per week and range of motion exercises once per day.

**Figure 2.**
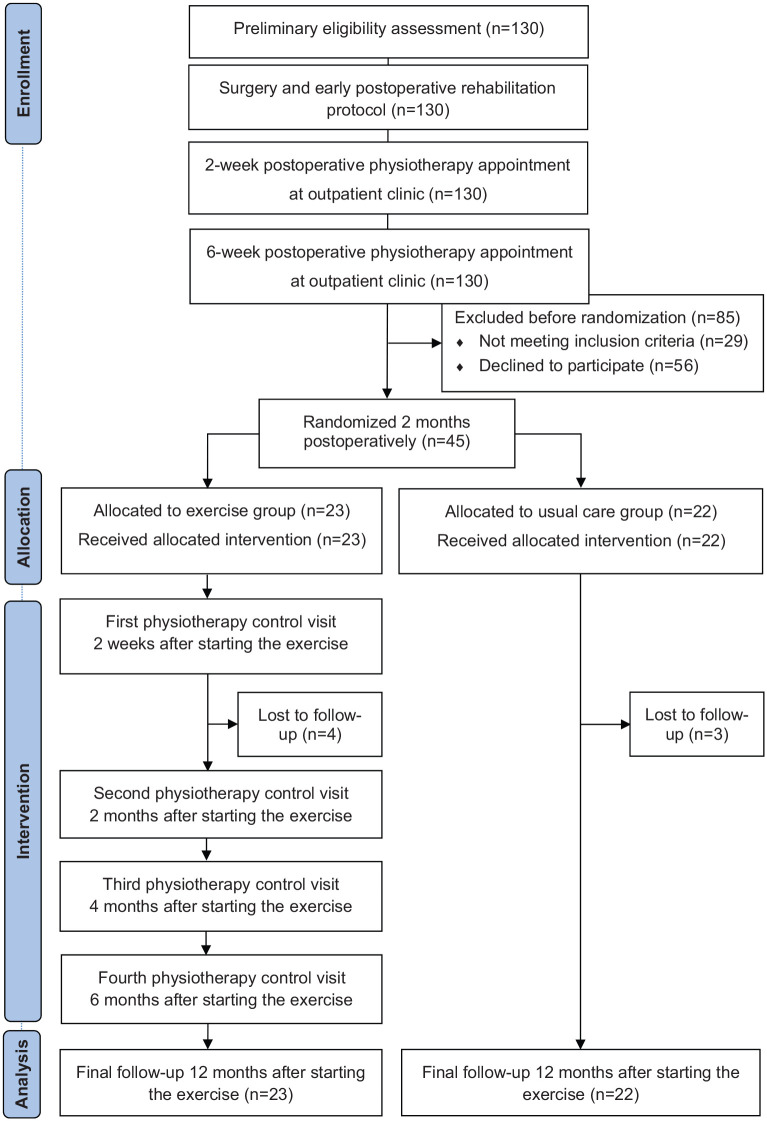
CONSORT diagram summarizing patient flow.

Two months after the operation, all patients visited the physiotherapist at the outpatient clinic of physical and rehabilitation medicine. Patients who met the inclusion criteria were stratified by gender and preoperative American Shoulder and Elbow Surgeons Standardized Shoulder Assessment Form^9^ indices (dichotomized as <50 points or ⩾50 points) and then randomized into either the progressive home exercise group or usual care control group (Figure 2) using sealed opaque envelopes and a computer-generated randomization list prepared using Medstat software.^10^ The randomization was performed by an independent research assistant not involved with the participants.

The experimental intervention was started two months after the operation. The patients randomized to the exercise group were given advice and instructions at the outpatient clinic on shoulder muscle strengthening exercises to be undertaken at home. The exercises were based on the best knowledge at the time.^11,12^ Patients were to perform the exercises three times per week for 12 months. Before starting, a physiotherapist demonstrated the exercises to individually each patient. The patients then executed the exercises and, if necessary, the physiotherapist corrected their technique and loading. Patients were lend the dumbbell with adjustable extra weights, and the dumbbell exercises were included in the program. The target for the patient was 10 repetitions at the beginning, gradually increasing to 15 repetitions. When patients were able to perform 15 repetitions, they were instructed to increase the weight by 0.5–1 kg. For males, the maximum loading was 18 kg and for females 11 kg. At intervention start, the exercises included wall push-ups, one-arm dumbbell rows, adduction of the shoulder with black rubber Thera-Bands^®^, internal and external shoulder rotations with dumbbell while lying on side, one-arm dumbbell presses in the supine position, shoulder front raise with dumbbell, bicep curls with dumbbell, abdominal crunches in supine position, and back extension while prone. Shoulder mobility exercises were continued to be performed daily. For the exercise group, training information was repeated in consecutive follow-ups.

All physiotherapy follow-ups in the exercise group were performed individually at the outpatient clinic of physical and rehabilitation medicine. The first was at two weeks after starting the exercises. On this occasion, the physiotherapist assessed the patient’s technique in conducting the exercises and, if necessary, corrected it. The next follow-up was six weeks later (i.e. at four months postoperatively) when, to ensure progression, additional exercises were added to the training program; dumbbell raises in a 45° horizontally adducted position, military push-ups, and triceps kickback with a dumbbell. The next follow-ups were at four and six months after starting the exercises (Figure 2). During these follow-ups, the physiotherapist ensured that the exercises were being performed in the right way. No additional exercises were added to the program.

The patients in the exercise group recorded their training frequency and possible pain and adverse events in a training diary, which were collected at the follow-ups. The patients in the control group did not receive any additional guidance or instructions beyond the usual postoperative exercise program to be undertaken at home.

At baseline, two months after the operation, all patients completed clinical and demographic questionnaires, and the American Shoulder and Elbow Surgeons Standardized Shoulder Assessment Form and Short-Form (SF)-36 Health Survey questionnaire.^13^ They filled in the questionnaires again at the end of the study, that is, 12 months after starting the exercises.

The American Shoulder and Elbow Surgeons Standardized Shoulder Assessment Form was used to assess self-reported disability.^14^ The scores were calculated from the self-evaluation form on shoulder-related physical function. The form contains two sections: pain experienced by the patient and a cumulative activities of daily living score. The pain score was calculated from the visual analogue scale (VAS) while the cumulative activities of daily living score is the sum of the scores for 10 activities of daily living items. Equal weight is given to both sections (50 points each), and thus, the total theoretical score is 100 points, with higher scores indicating better shoulder function.^9^ The American Shoulder and Elbow Surgeons Standardized Shoulder Assessment Form has been rigorously tested and proven to be a reliable, valid, and responsive outcome tool.^14–17^ It has also been validated in Finnish.^17^

Quality of life was assessed with the Short-Form 36 (SF-36) Health Survey.^18^ The SF-36 measures quality of life in eight dimensions: Physical Functioning, Role-Physical, Bodily Pain, General Health, Vitality, Social Functioning, Role Emotional, and Mental Health. Scores range from 0 to 100 in each dimension, with a higher score indicating better quality of health.^13^

Shoulder function was measured with objective muscle strength and range of motion measurements. The strength measurements were isometric and included grip force, full can test, and external and internal shoulder rotations. The strength measurements were assessed with a dynamometer (Ds Europe, Mod. 546QTD strain gauge, Milano, Italy) and analyzed with Protacon software (Jyväskylä, Finland). The shoulder range of motion measurements included active forward flexion, active as well as passive external and internal rotations, and passive horizontal adduction. Active and passive external rotations, passive internal rotation, and active forward flexion were assessed with a digital inclinometer (Baseline^®^; Fabrication Enterprises Inc., White Plains, NY, USA). Passive horizontal adduction was determined by measuring the distance between the epicondylus lateralis humeri and the acromion of the contralateral side using a tape measure. Active internal rotation was determined by measuring the distance between the C7 spinous process and the point where the thumb reached maximally behind the back using a tape measure. The visual analog scale (VAS: 0–100 mm) was used to measure whether the patients had felt pain during the strength and range of motion measurements.

Shoulder function data were collected at baseline (two months after the operation) and at 12 months thereafter. The outcome assessor and the research assistant who performed the randomization were blinded to the patients’ group allocation. However, owing to the research protocol, patients and physiotherapists were not blinded to assignment to the treatment group.

Data analyses were carried out by using IBM SPSS 22.0 software. The analysis was performed according to intention-to-treat (ITT) principles. Baseline demographic and clinical data were compared between groups using an independent samples *t*-test. The intensity of shoulder pain in the exercise group was analyzed by paired samples *t*-test. Between-group differences in changes in the SF-36 Health Survey dimensions, the American Shoulder and Elbow Surgeons Standardized Shoulder Assessment Form, and shoulder function measurements after the 12-month follow-up were analyzed using analysis of covariance (ANCOVA) with baseline values as covariates. The subjects who were lost to follow-up were included in the ITT analysis by imputing their missing values with the last observation carried forward (LOCF) method. The level of significance was *P* < 0.05.

## Results

The trial flow chart has been demonstrated in Figure 2. The total study sample comprised 32 men and 13 women. The baseline demographic and clinical characteristics are presented in Table 1.

**Table 1.** Baseline demographic and clinical characteristics of patients in the exercise group and control group.

	Exercise group *n* = 23	Control group *n* = 22
Male, *N* (%)	16 (70)	16 (73)
Age, years, mean (SD)	36 (11)	34 (10)
Height, cm, mean (SD)	174 (8)	175 (10)
Weight, kg, mean (SD)	75 (11)	80 (20)
ASES preoperative scoring, mean (SD)	67 (18)	72 (13)
Duration of shoulder pain before surgery, months, mean (SD)	62 (46)	69 (85)
Main shoulder symptoms before surgery, *N* (%)		
Instability	16 (70)	16 (73)
Pain	9 (39)	7 (32)
Numbness	0 (0)	1 (5)
Pathological lesions, *N* (%)		
Bankart lesion^a^	18 (78)	16 (73)
Anterior glenoid bone loss	7 (30)	4 (18)
SLAP lesion	10 (43)	9 (41)
Hill–Sachs lesion	13 (57)	14 (64)

SD: standard deviation; ASES: The American Shoulder and Elbow Surgeons Standardized Shoulder Assessment Form; SLAP: superior labral tear from anterior to posterior.

a Anterior–inferior capsulolabral lesion (with or without a bony lesion).

During the first six months of the intervention, eight patients (35%) in the exercise group were performing strength exercises and 12 patients (52%) were performing mobility exercises at the target level, which was at least twice per week. During the last six months, five patients (22%) in the exercise group were performing strength exercises and five patients (22%) were performing mobility exercises at the target level.

Mean intensity of shoulder pain (VAS: 0–100 mm) during the exercises decreased significantly from the baseline value of 10 (standard deviation (SD): 15) mm to the value of 2 (SD: 3) mm (*P* < 0.01) by the end of 12-month follow-up in the exercise group. No exercise-related adverse events were reported in the exercise group over the 12-month trial.

At the 12-month follow-up, both groups achieved a significant improvement in American Shoulder and Elbow Surgeons Standardized Shoulder Assessment Form and in the Physical Functioning, Role-Physical, and Bodily Pain dimensions of the SF-36, but there were no significant differences between the groups (Table 2).

**Table 2.** Baseline scores and changes in the ASES and SF-36 at 12-month follow-up.

	Baseline, mean (SD)		12 months, mean (SD)		Change to month 12, mean (95% CI)		*P* value between groups^a^
	Exercise	Usual care	Exercise	Usual care	Exercise	Usual care	
ASES (Scale 0–100)	78 (17)	79 (17)	95 (7)	92 (11)	16 (10 to 23)	13 (7 to 19)	0.203
SF-36 (Scale 0–100)							
Physical functioning	82 (21)	86 (16)	91 (21)	94 (11)	9 (3 to 15)	9 (3 to 15)	0.766
Role-physical	56 (39)	50 (40)	83 (36)	83 (30)	28 (10 to 47)	33 (15 to 51)	0.969
Role emotional	79 (35)	86 (29)	91 (25)	91 (23)	12 (3 to 21)	5 (−5 to 14)	0.406
Bodily pain	66 (23)	68 (24)	86 (17)	81 (25)	20 (11 to 30)	14 (4 to 24)	0.328
General health	78 (20)	82 (15)	77 (18)	82 (15)	−0.4 (−5 to 4)	−0.1 (−5 to 4)	0.632
Vitality	76 (17)	79 (14)	75 (19)	81 (12)	−1 (−4 to 3)	2 (−2 to 5)	0.239
Social functioning	85 (17)	96 (8)	95 (10)	97 (8)	10 (4 to 16)	1 (−6 to 7)	0.869
Mental health	80 (18)	84 (13)	84 (17)	87 (11)	4 (−1 to 7)	3 (−1 to 7)	0.898

ASES: American Shoulder and Elbow Surgeons Standardized Shoulder Assessment Form; SF-36: Short-Form 36 Health Survey; SD: standard deviation; CI: confidence interval.

a Adjusted by baseline values as covariates.

Both groups achieved a significant improvement in all aspects of range of motion and strength at the 12-month follow-up, but there were no significant between-group differences (Table 3).

**Table 3.** Baseline scores and changes at 12-month follow-up in range of motion and strength values of the operated shoulder.

	Baseline, mean (SD)		12 months, mean (SD)		Change to month 12, mean (95% CI)		*P* value
	Exercise	Usual care	Exercise	Usual care	Exercise	Usual care	Adjusted^a^
Range of motion							
Forward flexion^b^ (°)	150 (24)	158 (19)	173 (13)	179 (14)	23 (15 to 30)	22 (14 to 29)	0.269
External rotation, side^b^ (°)	44 (18)	48 (17)	67 (16)	73 (14)	23 (17 to 28)	25 (20 to 31)	0.219
90° external rotation^c^	51 (23)	58 (19)	86 (16)	88 (15)	34 (25 to 44)	31 (22 to 40)	0.786
90° internal rotation^c^	34 (14)	35 (12)	43 (14)	43 (9)	9 (5 to 13)	8 (4 to 12)	0.786
Internal rotation,^b^ C7 (mm)	294 (122)	305 (120)	188 (68)	195 (62)	−105 (−148 to −63)	−111 (−153 to −68)	0.854
Horizontal adduction^c^ (mm)	319 (55)	308 (49)	283 (39)	283 (40)	−37 (−56 to −17)	−26 (−45 to −6)	0.415
Muscle strength							
Grip strength (kg)	43 (12)	47 (13)	45 (12)	50 (14)	2 (0.1 to 4)	2 (0.3 to 4)	0.794
Full can (kg)	8 (4)	7 (3)	9 (3)	10 (3)	2 (1 to 3)	3 (2 to 4)	0.071
External rotation (kg)	9 (4)	9 (3)	11 (3)	11 (3)	2 (1 to 3)	2 (2 to 3)	0.248
Internal rotation (kg)	14 (6)	13 (6)	16 (6)	17 (7)	2 (0.1 to 3)	4 (2 to 5)	0.094

SD: standard deviation; CI: confidence interval.

a Baseline values as covariates.

b Active range of motion.

c Passive range of motion.

## Discussion

This study showed that a home-based additional exercise program with four physiotherapy control visits did not improve results compared to usual care. However, due to the small sample size and low level of training adherence, it was not realistic to expect major differences between the groups. Nevertheless, a significant decrease in self-reported disability and improvement in quality of life and shoulder function were observed in both groups at the 12-month follow-up after capsulolabral repair of the shoulder.

The findings of this study on postoperative recovery following capsulolabral repair are in line with those of previous studies.^3–5^ The present study compared the effects of two different types of rehabilitation programs, whereas previous studies have compared the effects of two identical rehabilitation programs that differed only in that one was closely supervised and the other unsupervised and conducted at home,^4^ and another one compared the effects of a conventional rehabilitation program and an early rehabilitation program that consisted strengthening exercises from the first postoperative day.^3^ Although early exercising after arthroscopic Bankart repair was found to be as safe as conventional rehabilitation in selected patients with a small Bankart lesion, the more intensive exercising in our study was designed to start more cautiously six week after operation to be in harmony with the healing process of the capsulolabral tissues. Excessive stress in the form of amount or timing may damage tissues or suture anchors.^19^

Our study results agree with the those of the three abovementioned postoperative exercise studies,^3–5^ all of which found no between-group differences in self-reported disability. Comparing our results to those of Kim et al.,^3^ both groups in our study achieved higher absolute American Shoulder and Elbow Surgeons Standardized Shoulder Assessment Form scores, although mean score changes by Kim et al. were significantly higher than those observed in our study. This is most probably because their baseline scores were calculated preoperatively, whereas we did not calculate our baseline scores until the randomization that took place two months postoperatively. A minimal clinically important difference in American Shoulder and Elbow Surgeons Standardized Shoulder Assessment Form scores of 6.4 points has been proposed for different shoulder pathologies.^15^ In our study, 15 patients (65%) in the exercise group and 17 patients (77%) in the control group achieved changes greater than 6.4 points. Six out of eight patients in the exercise group and five out of five patients in the control group who did not achieve a minimal clinically important difference exceeded 90 points already at baseline. This indicates a ceiling effect of the intervention on self-reported disability.

Comparison of quality of life between standard care and experimental rehabilitation following Bankart repair has previously been reported only by Damkjaer et al.^5^ Our study revealed similar results that postoperative rehabilitation improved, especially in physical health, that is, in Physical Functioning, Role-Physical, and Bodily Pain, whereas it had less or no effect on mental health, that is, Vitality, Mental Health, Role-Emotional, and Social Functioning. This is conceivable due to the fact that the scores of the mental health dimensions of the SF-36 were already high at baseline and therefore hard to improve on. It should be noted that we have previously measured same properties, that is, the dimensions of quality of life in comparing a 12-month home-based exercise program with usual care after rotator cuff repair.^8^ As in the present study, no between-group differences were found at follow-up in that study. The physical component summary score of the SF-36 also improved significantly, whereas the mental component summary score remained unchanged in both groups.^8^

Regaining pain-free shoulder range of motion is of great importance after a capsulolabral repair. In the present study, both groups achieved a significant improvement in all aspects of both active and passive ranges of motion at the 12-month follow-up. Damkjaer et al.^5^ reported similar results, although it is unclear whether they studied active or passive range of motion.

No previous studies have reported on changes in muscle strength after capsulolabral repair. In the present study, both groups attained significant improvements in each maximal isometric strength test. The biggest strength gain in the shoulder internal rotators is most probably due to the direction of movement in which the patient feels confident to contract and strong working muscles of pectoralis and subscapularis, both of which remain intact during capsulolabral repair. The smallest relative gain in hand-grip strength in turn may be explained by a reason that it was not a hand but shoulder injury, and the continued use of the hands in performing daily activities during the rehabilitation process, even if use of shoulder joint has been more limited.

Although this study could not demonstrate that a more intensive home-based exercise program would have additional benefit over usual care, the study findings do not exclude the possibility that some patients who recover poorly from capsulolabral repair could benefit from intensive rehabilitation coupled with additional physiotherapy visits. We demonstrated rather large individual variation in the outcomes in both groups. Thus, it is important to find those patients with delayed recovery who need more individualized rehabilitation. Future studies on patients after arthroscopic capsulolabral repair should focus on recognizing individual rehabilitation needs and finding effective methods to respond to those varying needs precisely and timely.

The main limitations of the current study were small study population and low training adherence. There may be several reasons for poor training adherence. First, there were no boosting follow-up visits during the last six months of the intervention. Second, six months after starting the exercises, the mean American Shoulder and Elbow Surgeons Standardized Shoulder Assessment Form score for the exercise group was approximately 91 points. Previously, a normative score of 92.2 (±14.5) for that measure has been considered as indicating an asymptomatic shoulder,^20^ and hence, the patients in the exercise group might have felt their shoulder was already well enough for them to reduce or discontinue the training. However, training adherence was low also in patients whose disability scores did not improve or improved only slightly. Another limitation is that we did not monitor the training frequency of the controls during the 12-month follow-up as it might have caused them to increase their physical activity, thereby affecting the results. Finally, this trial was reported more than 10 years after the study completion. Delay between completion and results reporting of this trial was due to the fact that our research team had limited resources, and we preferred to publish data first about rehabilitation after rotator cuff repaired patients^8^ from the broader study involving two different diagnostic groups. However, since there are limited number of studies that have been published on rehabilitation after capsulolabral repair, the findings of this study are still relevant.

The strengths of this study are that it was randomized and controlled with proper inclusion and exclusion criteria. This is also the first study to report the results of a 12-month exercise program for patients with capsulolabral lesions.

In conclusion, this study revealed that a more intensive exercise program after arthroscopic capsulolabral repair was no more effective than standard advice on exercise to be performed at home. The clinical implications of this study is that extra time involved in implementing additional physiotherapy control visits, at least along the lines followed in this study, cannot be recommended.
